# Population-level estimates of the proportion of *Plasmodium vivax* blood-stage infections attributable to relapses among febrile patients attending Adama Malaria Diagnostic Centre, East Shoa Zone, Oromia, Ethiopia

**DOI:** 10.1186/s12936-017-1944-3

**Published:** 2017-07-27

**Authors:** Lemu Golassa, Michael T. White

**Affiliations:** 1 Aklilu Lemma Institute of Pathobiology, Addis Ababa University, Addis Ababa, Ethiopia; 2 MRC Centre for Outbreak Analysis and Modelling, Department of Infectious Disease Epidemiology, Imperial College London, Norfolk Place, London, W2 1PG UK; 3 Division of Population Health and Immunity, The Walter and Eliza Hall Institute, 1G Royal Parade, Melbourne, VIC 3052 Australia

**Keywords:** Malaria, *P. vivax*, Relapse, Hypnozoites, Ethiopia

## Abstract

**Background:**

Malaria is ranked as the leading communicable disease in Ethiopia, where *Plasmodium falciparum* and *Plasmodium vivax* are co-endemic. The incidence of *P. vivax* is usually considered to be less seasonal than *P. falciparum*. Clinical cases of symptomatic *P. falciparum* exhibit notable seasonal variation, driven by rainfall-dependent variation in the abundance of *Anopheles* mosquitoes. A similar peak of clinical cases of *P. vivax* is usually observed during the rainy season. However, the ability of *P. vivax* to relapse causing new blood-stage infections weeks to months after an infectious mosquito bite can lead to substantial differences in seasonal patterns of clinical cases. These cannot be detected with currently available diagnostic tools and are not cleared upon treatment with routinely administered anti-malarial drugs.

**Methods:**

A health- facility based cross-sectional study was conducted in Adama malaria diagnostic centre from May 2015 to April 2016. Finger-prick blood samples were collected for thin and thick blood film preparation from participants seeking treatment for suspected cases of febrile malaria. Informed consent was obtained from each study participant or their guardians. Seasonal patterns in malaria cases were analysed using statistical models, identifying the peaks in cases, and the seasonally varying proportion of *P. vivax* cases attributable to relapses.

**Results:**

The proportion of patients with malaria detectable by light microscopy was 36.1% (1141/3161) of which *P. vivax*, *P. falciparum*, and mixed infections accounted for 71.4, 25.8 and 2.8%, respectively. Of the febrile patients diagnosed, 2134 (67.5%) were males and 1919 (60.7%) were urban residents. The model identified a primary peak in *P. falciparum* and *P. vivax* cases from August to October, as well as a secondary peak of *P. vivax* cases from February to April attributable to cases arising from relapses. During the secondary peak of *P. vivax* cases approximately 77% (95% CrI 68, 84%) of cases are estimated to be attributable to relapses. During the primary peak from August to October, approximately 40% (95% CrI 29, 57%) of cases are estimated to be attributable to relapses.

**Discussion:**

It is not possible to diagnose whether a *P. vivax* case has been caused by blood-stage infection from a mosquito bite or a relapse. However, differences in seasonal patterns of *P. falciparum* and *P. vivax* cases can be used to estimate the population-level proportion of *P. vivax* cases attributable to relapses. These observations have important implications for the epidemiological assessment of *vivax* malaria, and initiating therapy that is effective against both blood stages and relapses.

**Electronic supplementary material:**

The online version of this article (doi:10.1186/s12936-017-1944-3) contains supplementary material, which is available to authorized users.

## Background

*Plasmodium vivax* is the most widely distributed species of malaria in the world, causing an estimated 8.5 (6.6–10.8) million clinical cases per year [1]. Across the globe, there are about 3 billion people at risk of *P. vivax* infection [2]. Despite this large burden of disease, *P. vivax* is often overlooked compared to *Plasmodium falciparum,* which is responsible for the majority of malaria-associated deaths, predominantly in young children and pregnant women in sub-Saharan Africa. Ethiopia together with India, Indonesia, and Pakistan accounts for more than 80% of the global *P. vivax* burden [3].

About 75% of the landmass of Ethiopia is either malarious or potentially malarious and an estimated total of 68% of the Ethiopian population live at altitudes below 2000 m and are considered to be at risk of malaria. In Ethiopia, *P. falciparum* and *P. vivax* account for about 60 and 40% of all malaria cases, respectively [4]. Malaria transmission is generally seasonal and unstable with substantial spatial and inter- and intra-annual variation. The seasonality is attributed to variation in the vector population (due to variation in the suitability of climatic conditions for vector breeding) across different months of the year. In general, two main seasons for the transmission of malaria are known in Ethiopia: the major transmission season from September to December following the main rainy season from June to August and the minor transmission season from April to May following the shorter rainy season in March, although the transmission seasons generally vary from region to region depending on climatic conditions such as temperature, rainfall, and humidity [5]. In some parts of the country where temperature and rainfall conditions are nearly always favorable the transmission of malaria occurs throughout the year. In Ethiopia, most malaria control interventions are initiated during wet seasons. The co-existence of both *P. falciparum* and *P. vivax* in Ethiopia creates challenges for prevention, control and elimination of the disease. *Anopheles arabiensis* is the main malaria vector although *Anopheles pharoensis*, *Anopheles funestus* and *Anopheles nili* also transmit malaria [6].

The interruption of *P. vivax* transmission is not easy because of its unique biological features, most notably, the ability for relapses to cause new blood-stage infections weeks to months after the initial infectious mosquito bite [7]. In contrast to *P. falciparum*, which has only sporozoite induced infections, *P. vivax* blood-stage parasitaemia can arise from either mosquito-borne sporozoites or liver-stage hypnozoites. Hypnozoites arise from *P. vivax* sporozoites that invade liver-stage hepatocytes and lie dormant for weeks to years before activating to cause relapses [8]. Assigning the exact source of any given blood stage infection (parasitaemia) observed in endemic settings is usually impossible, but the force of infection attributable to sporozoites versus hypnozoites may still be inferred at a population level [8–10].

Relapse may well be the predominant origin of most *P. vivax* clinical attacks throughout the endemic areas. Failure to systematically attack the hypnozoite reservoir of *P. vivax* not only results in repeated clinical attacks and serious illness, but also creates further opportunities for transmission to others [11]. The time to first relapse after primary infection has been observed to be approximately 3 months in Ethiopia [12]. When the transmission season ceases as a consequence of decline and/or absence of the mosquito population, most *vivax* malaria cases in the dry season are likely due to hypnozoite reactivation. Although hypnozoites do not contribute to clinical disease until activated, these dormant stages of the parasite ultimately play a vital role in sustaining transmission as they are refractory to blood-stage antimalarial drugs and interventions to reduce transmission. Furthermore, hypnozoites also ensure the ability of *P. vivax* to survive in climatic conditions that cannot sustain *P. falciparum* transmission [13, 14].

Representing an important reservoir for new infections, *P. vivax* hypnozoites are less susceptible to conventional malaria control interventions such as insecticides, bed nets, diagnostic methods, and almost all chemoprophylactic or chemotherapeutic interventions [7]. Only one class of drugs, 8-aminoquinolines, has been proven to successfully prevent relapses, with primaquine being the only drug from this class currently licensed for use. In most endemic countries chloroquine (CQ) and primaquine (PQ) have been used as the first-line therapies for *P. vivax* infections except where *P. vivax* is resistant to CQ [15]. There are currently insufficient measures in place to ensure the safe delivery of PQ within the context of glucose-6-phosphate dehydrogenase deficiency (G6PDd) risk [16]. As PQ may cause a life threatening haemolytic anemia in patients with G6PD deficiency, it is not recommended to patients whose G6PD deficiency status is not known.

Estimates of the force of blood-stage infections arising from primary infections and relapses are important for designing intervention strategies although their relative contributions in endemic settings are not well established. The seasonality of *P. vivax* primary infections can be estimated from the seasonality of *P. falciparum* infections since they are transmitted by the same vectors. A statistical model has been developed to estimate the incidence and seasonality of *P. vivax* primary infection and relapse [17], given longitudinal data with genotyped samples. The seasonal pattern of *P. falciparum* infections provides approximate estimates of the seasonal pattern of *P. vivax* primary infections. In co-endemic settings, *P. falciparum* is often the first species to show a decline in incidence during successful control campaigns with *P. vivax* generally being slower to respond to the same interventions. As a result significant reductions in *P. vivax* case burden may only become evident a few years after decreases of *P. falciparum*, due to the hidden reservoir of silent *P. vivax* hypnozoites [18]. It is uncertain how relapses contribute to the burden of *P. vivax* malaria in areas of seasonal transmission settings. Effective control strategies for *P vivax* will be aided by improving our understanding of the proportion of clinical cases of *P. vivax* caused by relapses. The objective of this study was to estimate the proportion of clinical cases of *P. vivax* attributable to relapses in Adama malaria diagnostic centre, East Shoa Zone, Oromia, Ethiopia.

## Methods

### Study area

The study was conducted in Adama malaria diagnostic centre, East Shoa Zone, Oromia, Ethiopia from 15 May 2015 through 15 April 2016. The health centre is dedicated to malaria diagnosis as it does not perform any other types of diagnosis. Adama town (8°33′N 39°17′E) is located 100 km from Addis Ababa, Ethiopia. People from the Adama town and the surrounding rural areas preferentially use this laboratory for malaria diagnosis over hospitals and other surrounding health centres. The population of Adama within reach of the health centre is approximately 324,000. As individuals can obtain malaria diagnosis and treatment at other facilities, this can be considered as an upper limit on the population denominator. The laboratory technicians at this centre are WHO certified microscopists. In the study area, malaria transmission is seasonal like many endemic regions of Ethiopia and both *P. falciparum* and *P. vivax* co-exist. Although malaria transmission varies from year to year depending on rainfall patterns, the major transmission season in the study area usually runs from August through December with a minor transmission season from May through June.

### Sample collection

Self-reporting febrile patients seeking malaria diagnosis were included in this study. Demographic characteristics of individuals presenting at Adama malaria diagnostic centre were systematically registered on the logbook (routine procedures). Blood samples were obtained through finger prick method for thick and thin smears. The slides were stained with a 10% Giemsa solution to be examined under 100× microscopes for the presence of malaria parasites. The slides were independently read by two skilled microscopists. Ethiopia adopted species-specific malaria treatment policy making species identification very crucial for proper prescription of the right anti-malarial drugs. Those febrile individuals who were negative for malaria were advised to undertake further diagnosis for their febrile illness at hospitals or elsewhere. Patients with confirmed cases of *P. vivax* received an unsupervised 3-day treatment of chloroquine (total 25 mg/kg) without primaquine, while cases of confirmed *P. falciparum* and mixed infections (*P. falciparum* and *P. vivax*) received artemether–lumefantrine.

### Ethics

Ethical clearance for this study was obtained from Aklilu Lemma Institute of Pathobiology Institutional Review Board. Informed consents were obtained from participating individuals.

### Data analysis

The data were entered into an excel sheet and analysed using stata/MP 13.0 version. The seasonal variation in *P. falciparum* cases can be described by the following functional form [14, 19].

1 $$Pf\left( t \right) = Pf_{0} \left( {c + \left( {1 - c} \right)\frac{\pi }{{B\left( {\tfrac{1}{2},\;\kappa + \tfrac{1}{2}} \right)}}\left( {\frac{{1 + \cos \left( {\tfrac{2\pi }{12}\left( {t - \theta } \right)} \right)}}{2}} \right)^{\kappa } } \right)$$

where *Pf*_*0*_ is the average number of *P. falciparum* cases per month, *c* is the ratio between cases at the lowest point of the dry season and the peak of the wet season, *κ* is a parameter determining the seasonality (larger values of *κ* correspond to higher degrees of seasonality), and θ determines the timing of the peak of seasonality. *B* is the beta function which assures the seasonal function is appropriately normalized. *It is assumed* that the incidence of cases of *P. vivax* caused by primary infections is proportional to the incidence of *P. falciparum* cases [17], and that the incidence of *P. vivax* cases caused by relapses is proportional to the incidence of *P. falciparum* cases δ months ago. The incidence of *P. vivax* can then be described by the following formula.

2 $$Pv\left( t \right) = \alpha Pf\left( t \right) + \beta Pf\left( {t - \delta } \right)$$

**Fig. 1:**
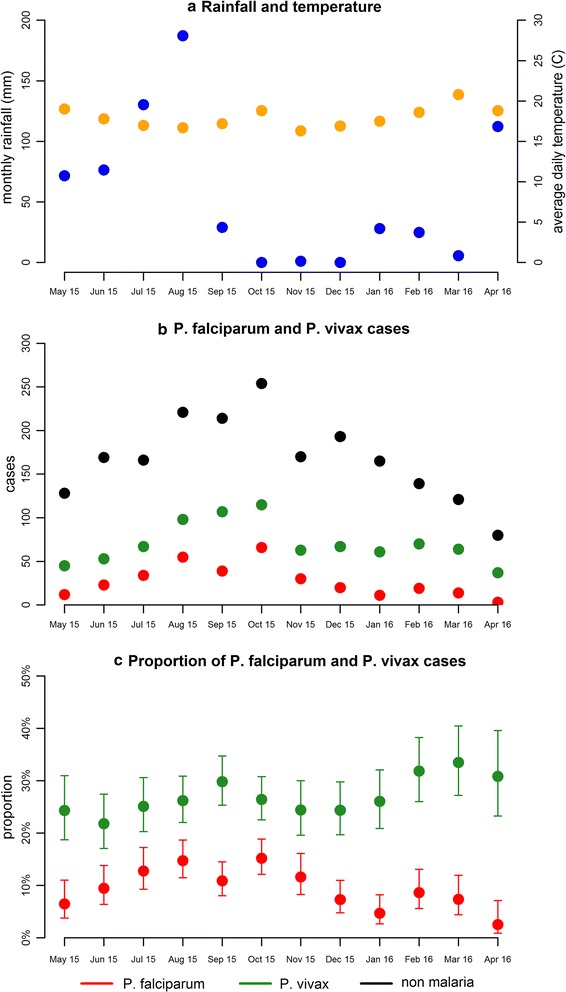
Seasonal variation in *P. falciparum* and *P. vivax* cases. **a** Total monthly rainfall (mm) in *blue*, and the median of the daily average temperature in degrees Celsius in *orange*. **b** Monthly cases of *P. falciparum*, *P. vivax* and non-malaria cases. **c** The proportion of suspected monthly cases confirmed as either *P. falciparum* or *P. vivax*

The models for seasonal variation in *P. falciparum* and *P. vivax* cases described in Eqs. (1) and (2) are jointly fitted to the data on malaria cases. Importantly, this model can be fitted to the data in two different formats: seasonal variation in the total cases of *P. vivax* and *P. falciparum* (Fig. 1b); and seasonal variation in the proportion of cases of *P. vivax* and *P. falciparum* (Fig. 1c). The statistical model was fitted separately to these two formats of the data: (a) data on proportion of cases using a Binomial likelihood; and (b) data on total cases using a normal likelihood. The models were fitted in a Bayesian framework using improper uniform priors.

## Results

A total of 3161 patients visited Adama malaria diagnostic centre from May 2015 through April 2016. The proportion of patients with malaria detectable by light microscopy was 36.1% (1141/3161). *P. vivax* was the predominant species (71.4%) identified in the study area. Of the study participants 67.5% were males and 60.7% were urban residents. Age group 6–15 years had the highest incidences of malaria (49.5%) with the age group greater than 45 years having the lowest incidence (26.0%). Pattern of *P. vivax* infections by age across the 12 months of the study period is presented in Additional file 1. The demographic characteristics of the study participants are shown in Table 1. For both *P. falciparum* and *P. vivax*, the peak of malaria was from August through December. The number of people presenting at the health facility and monthly malaria clinical cases due to *P. falciparum* and *P. vivax* is summarized in Table 2. Parameters of the statistical model used to analyse data on proportion of cases and data on total cases are presented in Table 3.

**Table 1:** Demographic characteristics of the study participants

Variables	Number examined	Number positive (%)
Age groups (in years)		
0–5	215	86 (40.0)
6–15	418	207 (49.5)
16–25	941	340 (36.1)
26–35	724	246 (34.0)
36–45	455	156 (34.3)
46–55	217	60 (27.6)
56–65	121	34 (28.1)
66–75	56	11 (19.6)
>75	14	1 (7.1)
Residence		
Urban	1919	565 (29.4)
Rural	1242	576 (46.4)
Sex		
Male	2134	822 (38.5)
Female	1027	329 (32.0)

**Table 2:** Data on recorded cases of clinical malaria

Month	People presenting at health centre	Clinical malaria case		
		*P. vivax* ^a^	*P. falciparum* ^a^	*P. vivax* and *P. falciparum*
May 2015	185	45	12	0
Jun 2015	243	53	23	2
Jul 2015	267	67	34	0
Aug 2015	374	98	55	0
Sep 2015	359	107	39	1
Oct 2015	435	115	66	0
Nov 2015	258	63	30	5
Dec 2015	275	67	20	5
Jan 2016	234	61	11	3
Feb 2016	220	70	19	8
Mar 2016	191	64	14	8
Apr 2016	120	37	3	0

^a^Includes mixed infections

**Table 3:** Estimated parameters of the statistical model. Parameters are presented as median and 95% credible intervals of the estimated posterior distribution

Parameter	Description	Data analysed	
		Proportions	Total cases
*Pf*_*0*_	Mean proportion/number of *P. falciparum cases per month*	9.4% (8.4, 10.4%)	27 (21, 33)
c	Ratio of cases in dry season to wet season	0.69 (0.55, 0.84)	0.56 (0.28, 0.79)
κ	Seasonal shape parameter	2.3 (1.5, 4.3)	3.0 (1.7, 7.0)
θ	Offset for seasonal peak (0 = May 2015)	4.9 (4.4, 5.4)	5.1 (4.6, 5,5)
α	Primary *P. vivax* cases relative to *P. falciparum* cases	1.17 (0.77, 1.49)	1.91 (1.19, 2.65)
β	Relapses *P. vivax* cases relative to *P. falciparum* cases	1.72 (1.35, 2.12)	0.71 (0.13, 1.39)
δ	Lag of relapse cases compared to primary cases	5.9 (5.2, 6.5)	4.8 (0.8, 10.8)
σ	Standard deviation of case numbers	–	10.3 (7.4, 16.3)

**Fig. 2:**
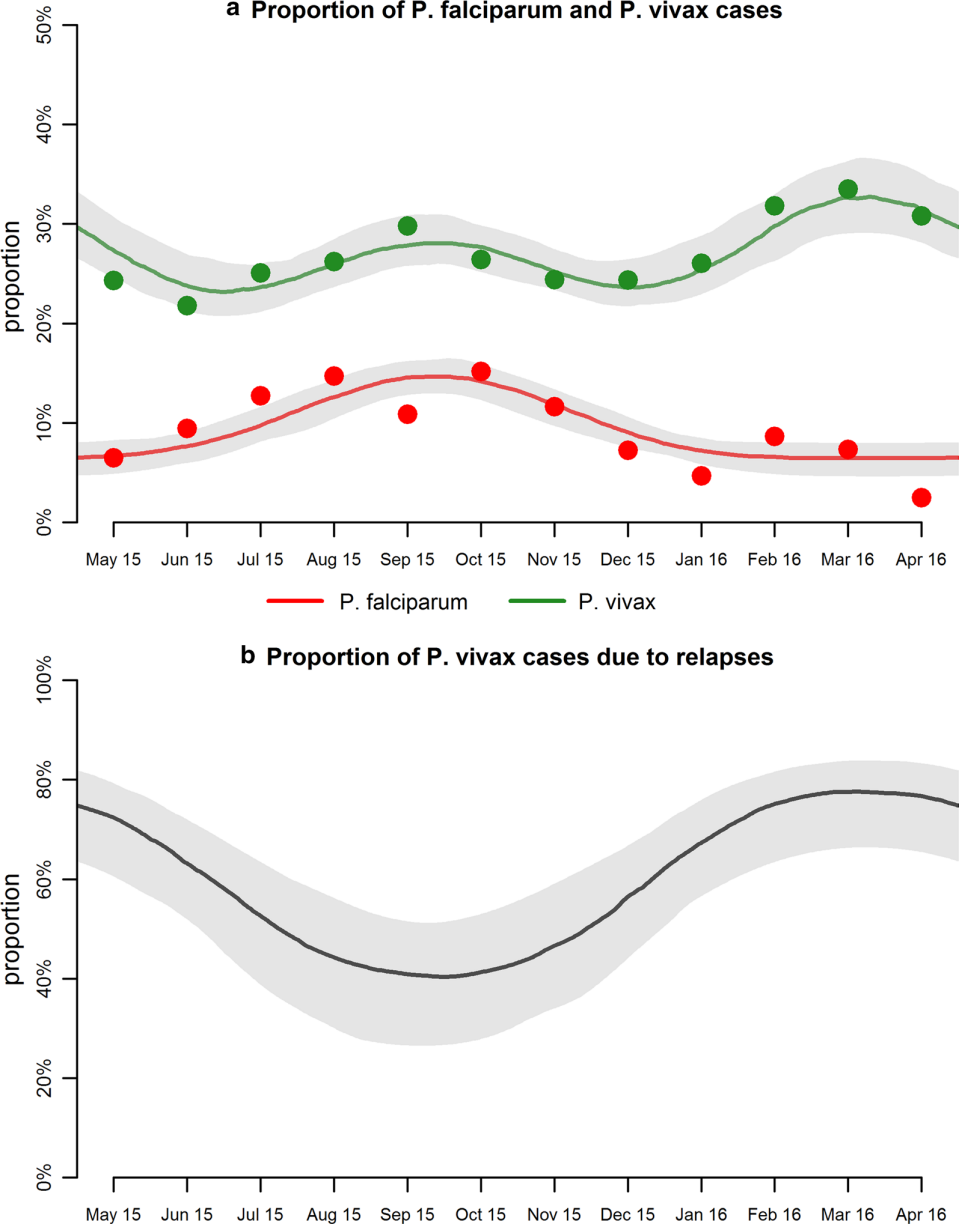
Results of fitting statistical models to the seasonal proportion of *P. falciparum* and *P. vivax* cases. The *solid line* shows the best fit and the *shaded regions* denote the 95% credible intervals

Figure 2 shows the results of fitting statistical models to the data on the proportion of cases that are due to either *P. falciparum* or *P. vivax*. The model identifies the primary peak in *P. falciparum* cases from August to October seen in the data. The model also identifies the peak of cases of *P. vivax* attributable to primary infection seen in the data from August to October, as well as a secondary peak from February to April attributable to cases arising from relapses. Examining the seasonal variation in clinical cases of *P. vivax* attributable to relapses, *it was estimated* that during the secondary peak in February to April 77% (95% CrI 68, 84%) of cases are attributable to relapses. During the primary peak from August to October, it is estimated that 40% (95% CrI 29, 57%) of cases are attributable to relapses.

**Fig. 3:**
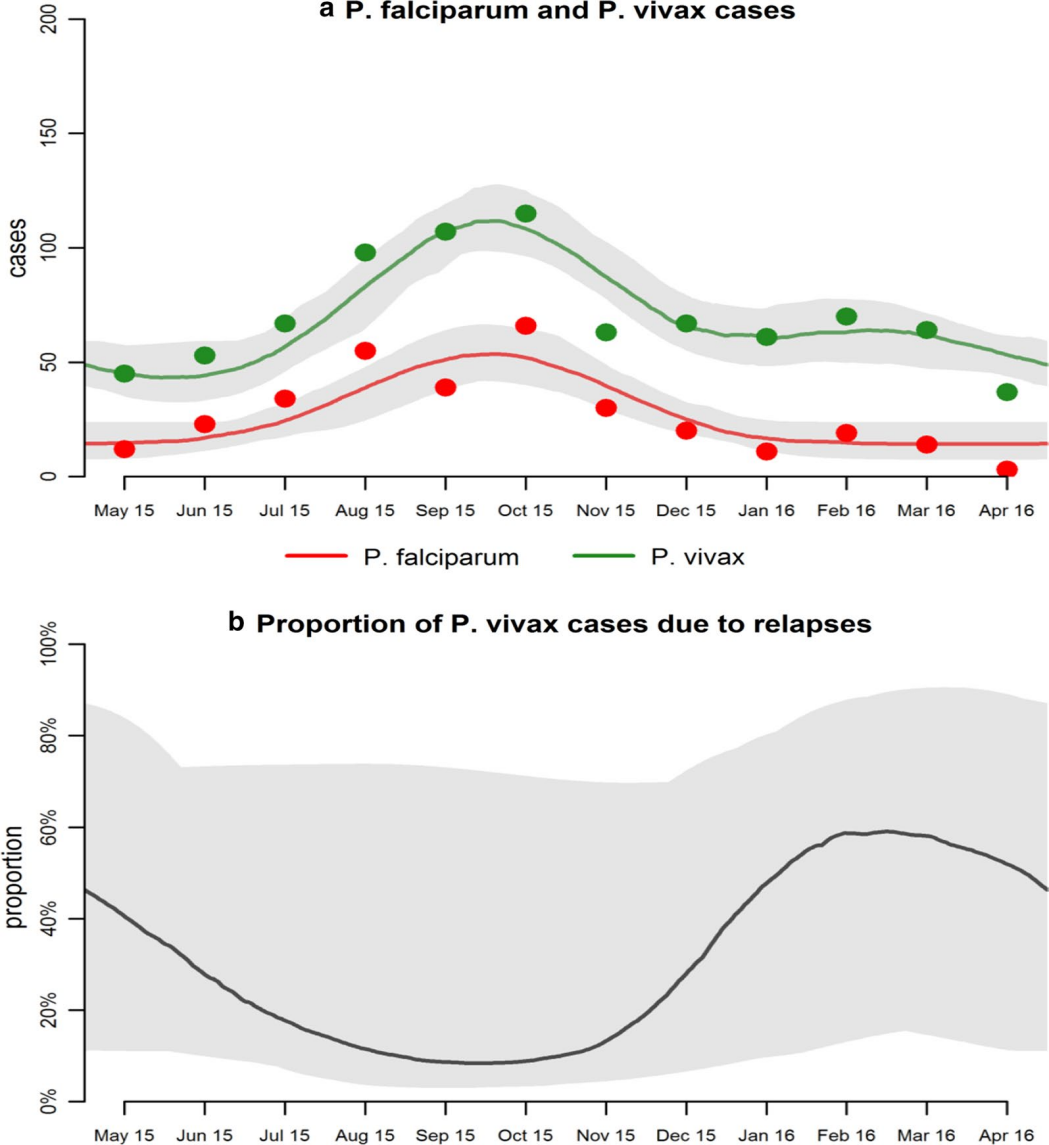
Results of fitting statistical models to the seasonal numbers of *P. falciparum* and *P. vivax* cases. The *solid line* shows the best fit and the shaded regions denote the 95% credible intervals

Figure 3a shows the results of fitting the model for seasonal variation to the data on total case numbers of *P. vivax* and *P. falciparum*. For both species, the model predicts a peak during September to October. A minor secondary peak of *P. vivax* cases is also predicted from February to March. It should also be noted that the data suggest a minor secondary peak in *P. falciparum* cases during this period as well.

Figure 3b shows the estimated proportion of clinical cases of *P. vivax* attributable to relapses. Although the line of best fit does suggest substantial seasonal variation in the cases attributable to relapses, the credible intervals are very wide, spanning most of the range from 0 to 100%. This suggests that the model and data interpreted in this manner does not have sufficient statistical power to identify the seasonal variation in relapses.

## Discussion

In this study, the proportion of individuals with malaria was 36.1% (1141/3161) as diagnosed by light microscopy. The highest incidence of malaria was during the peak malaria transmission season for the study site from August to October. Malaria cases were highest among age group 6–15 years (49.5%), followed by the <5 age group (40.0%) (Table 1). It is apparent that most malaria mortality and morbidity in endemic settings occurs in children under 5 years of age. Malaria cases were highest among rural (46.4%, 576/1242) residents compared to urban residents (29.4%, 565/1919). Inhabitants of urban areas may have lower exposure to mosquito bites than rural inhabitants and hence have lower malaria incidences. Males had a slightly higher proportion of confirmed malaria cases (38.5%) than females (32.0%). The presence of sex-specific occupational variation in the study area (mainly outdoor activities in males and indoor activities in females) may account for variable exposure to mosquito bites and hence lower incidence of malaria cases in the latter.

The incidence of *P. vivax* appears to decrease more slowly than that of *P. falciparum* in areas where both species coexist [1]. As the incidence of malaria is reduced, the proportion of all cases due to *P. vivax* increases and predominates in the area. During the peak transmission season, both falciparum and vivax malaria cases appear at health facilities. As the transmission season ceases following the unfavourable weather conditions for vector breeding, *P. falciparum* cases decline while *vivax* malaria cases continue appearing at health facilities because of latent liver-stage infections. Thus, the appearance of *P. falciparum* cases after a pause of transmission signals the beginning of the next malaria transmission season. The continuation of *P. vivax* cases even after the primary transmission season underscores the need for effective intervention strategies against liver-stage parasites in Ethiopia. However, with currently available technology, it is not possible to diagnose latent liver stages. Another point worth noting is the complaints of treatment fatigue by individuals receiving treatment for *P. vivax*. As Ethiopia’s national treatment guideline does not allow PQ for the radical cure, the inevitable recurrence of *P. vivax* after CQ therapy has made people less interested to seek health facilities. *Plasmodium vivax* cases appearing at health facilities during the non-transmission season likely arise from reactivation of dormant liver-stages. These individuals had *P. vivax* primary infections sometime ago during the previous transmission seasons and did not receive PQ for radical cure for their illness. The infections that arise from the reactivation of dormant liver-stage in the dry season may not be transmitted from infected individuals to uninfected ones as the vectors responsible for active transmission are non-existent due to the unsuitability of the weather conditions in the dry season. Indeed, in the transmission season *P. vivax* cases are presumed to be both from new mosquito infections and relapse unlike the *vivax* malaria episodes in the dry season that emanate from the latter. As a consequence, clearance of acute vivax malaria requires two distinct classes of drugs: blood schizonticide(s) (to terminate the acute attack), and hypnozoitocide(s) (to prevent subsequent acute attacks) [15, 16]. Prevention of infections arising from relapse is challenging as it is less susceptible to the conventional malaria control interventions such as insecticides, bed nets, diagnostic methods and almost all chemotherapeutic interventions [7]. The distinct biological characteristics of *P. vivax* present challenges for its control and elimination. Insecticide-treated mosquito nets (ITNs) and indoor residual spraying (IRS), for instance, are not always as effective against *P. vivax* as they are against *P. falciparum*. The reservoir of *P. vivax* infection in the human liver stage (hypnozoites), can result in cases occurring without the bite of infectious vectors.

Several historical studies have shown that *P. falciparum* and *P. vivax* substantially contribute to malaria morbidity in Ethiopia, in relative proportions of approximately 60 and 40% [20–22], respectively, although their relative proportions vary both temporally and spatially. The predominance of *P. vivax* cases in the study site is consistent with a globally reported epidemiological trend in *P. vivax* and *P. falciparum* co-endemic regions where the proportion of *P. vivax* cases increases as total malaria cases are reduced via malaria control interventions. However, to definitively show this for the study site in Adama would require additional data from before the roll out of insecticide treated nets.

The statistical model applied here utilizes routinely collected data on monthly cases of *P. falciparum* and *P. vivax* to estimate the proportion of *P. vivax* cases attributable to relapses. Notably, *the results of this study* are consistent with the findings from an analysis of data from cohorts of Papua New Guinean children that indicate that approximately 81% (95% CI 42, 94%) of clinical cases of *P. vivax* are attributable to relapses [10] and that the proportion of *P. vivax* infections attributable to relapses is highest in the dry season [17]. However, it should be noted that there are some challenges in interpreting the data due to the seasonality in non-malaria cases reporting to the health centre (Fig. 1b). The seasonal variation in non-malaria cases could be due to (1) seasonally varying incidence of other infectious diseases; (2) sub-microscopic symptomatic cases of malaria; or (3) individuals systematically over-accessing resources at the health centre during the malaria transmission season. Given these uncertainties, it is unclear whether the true seasonal pattern of cases of clinical malaria in the population as a whole is better represented by the proportion of cases of confirmed *P. vivax* and *P. falciparum*, or by total case numbers.

When the statistical model *is applied* to the data on proportion of cases with *P. vivax* there is a clear secondary peak of *P. vivax* cases in February to March (Fig. 2a). However when the statistical model *is applied* to total *P. vivax* cases, there is much less evidence for a secondary peak of *P. vivax* cases from February to March (Fig. 3a). In both cases, the proportion of *P. vivax* cases attributable to relapses is predicted to be highest during February to March, although there is not enough signal for a significant pattern when data are analysed using total cases.

Seasonal patterns in clinical cases of *P. vivax* may not accurately reflect seasonal patterns in the incidence of *P. vivax* blood-stage infections. The incidence of *P. vivax* relapses is believed to be highest immediately after the initial infectious mosquito bite [23], but possibly with a delay of a few weeks [17, 24]. However, *the model* fit to the data suggests a delay of 5–6 months between exposure during the peak malaria transmission season and the incidence of clinical cases of *P. vivax* attributable to relapses. This could be due to the relapse phenotype of Ethiopian *P. vivax* parasites [25, 26], or may be a consequence of induced immune responses. An adaptive antibody response targeting the primary blood-stage *P. vivax* infection may provide protection against high density parasitaemia and clinical episodes arising from relapses, particularly if the relapse and primary infection are clonal [27]. Indeed, it is plausible that a relapse may only cause a clinical case of *P. vivax* if: (1) it occurs after a sufficient duration such that short-term adaptive responses generated by the primary infection have waned [28, 29]; and (2) the relapsing parasite is not a full sibling of the parasites that caused the primary infection [30].

It is apparent that the treatment of *P. vivax* patients with primaquine radical cure may reduce relapse associated morbidity. However, many malaria endemic countries have not mandated routine glucose-6-phosphate dehydrogenase (G6PD) testing before initiating PQ for radical cure of patients infected by *P. vivax* malaria. This has led to the absence of PQ prescription or inconsiderate prescription and administration of PQ to *P. vivax* patients without being concerned about patients’ G6PD status and associated complications [31]. Indeed, the single greatest obstacle to PQ prescription is the rational fear of complications associated with this drug in G6PD deficient patients. Towards this end, a study conducted in the Gambella region of Ethiopia showed the presence of G6PD deficiency in Nilotic ethnic group [32] that represents less than 1% of the total population. Further assessments of G6PD deficiency nationwide will help abate relapse-associated morbidity by providing PQ for radical cure.

Data on the incidence of *P. falciparum* and *P. vivax* cases are often routinely collected at various levels of the public health system. By comparing the seasonal patterns in *P. falciparum* and *P. vivax* cases, this method suggests that after the primary malaria transmission in Adama, Ethiopia up to 77% (95% CrI 68, 84%) of *P. vivax* cases are attributable to relapses. This provides an indication of the burden of *P. vivax* malaria that can be prevented if more individuals are treated with primaquine during the wet season, either following a primary case of *P. vivax* in the wet season or by mass treatment of asymptotic individuals in the wider population. This is the first study in Ethiopia to address population-level estimates of the proportion of *P. vivax* clinical cases attributable to relapses among febrile patients. As relapses present a challenge to control and elimination programmes, the hypnozoite needs to be the focus of specific intervention programmes for the realization of malaria elimination. These observations have important implications for the epidemiological assessment of *vivax* malaria, and initiating therapy that is effective against both blood stages and relapses.

## Conclusion

This study estimated the proportion of clinical cases of *P. vivax* attributable to relapses, which has important implications for the epidemiological assessment of *vivax* malaria and initiating therapy that is effective against both blood stages and relapses. The study provides an indication of the burden of *P. vivax* malaria that can be prevented if more individuals are treated with primaquine during the wet season, either following a primary case of *P. vivax* or by mass treatment of asymptomatic individuals in the wider population. The model suggest that 70% of *P. vivax* infections are suggested to have arisen from relapse during February to April, while 40% of *P. vivax* cases resulted from relapse during August to October 2015. The model also shows the occurrence of the relapse phenotype of Ethiopian *P. vivax* parasites with a delay of 5–6 months between exposure during the peak malaria transmission season and the incidence of clinical cases of *P. vivax* attributable to relapses. Undoubtedly, the successful control and elimination of *P. vivax* malaria calls for specific and additional interventions, particularly against the liver stage of the parasite.

## Additional file

**Additional file 1.** Pattern of *P. vivax* infections by age across the 12 months of the study.
