# Supplementary material for: Population-level estimates of the proportion of Plasmodium vivax blood-stage infections attributable to relapses among febrile patients attending Adama Malaria Diagnostic Centre, East Shoa Zone, Oromia, Ethiopia
Source: Malar J. 2017 Jul 27;16:301. doi: 10.1186/s12936-017-1944-3 (PMC5530918; doi:10.1186/s12936-017-1944-3)
Supplement: Supplementary file 1 — Additional file 1. Pattern of P. vivax infections by age across the 12 months of the study. [file 12936_2017_1944_MOESM1_ESM.doc]

**Supplementary Table 1**

Pattern of *P. vivax* infections by age across the 12 months of the study

| Study months | No. +ve/  No. examined | *P. vivax* and mixed infections by age across the 12 months | | | | | | | | Total |
| --- | --- | --- | --- | --- | --- | --- | --- | --- | --- | --- |
|  |  | **0-5**  No.+ve/exam | **6-15**  No.+ve/exam | **16-25**  No.+ve/exam | **26-35**  No.+ve/exam | **36-45**  No.+ve/exam | **46-55**  No.+ve/exam | **56-65**  No.+ve/exam | **>65**  No.+ve/exam |  |
| 15-May | 45/185 | 0/8 | 4/17 | 13/53 | 13/52 | 8/30 | 6/17 | 0/3 | 1/5 | 45 |
| 15-Jun | 53/243 | 1/9 | 6/24 | 15/69 | 14/63 | 10/46 | 4/18 | 3/11 | 0/3 | 53 |
| 15-Jul | 67/267 | 4/9 | 12/29 | 26/86 | 17/70 | 5/35 | 1/24 | 1/7 | 1/7 | 67 |
| 15-Aug | 98/347 | 6/17 | 18/54 | 27/120 | 24/80 | 15/60 | 4/22 | 2/10 | 2/11 | 98 |
| 15-Sep | 107/359 | 10/34 | 26/56 | 39/96 | 19/71 | 9/45 | 2/29 | 2/21 | 0/7 | 107 |
| 15-Oct | 115/435 | 12/35 | 29/64 | 35/127 | 13/91 | 17/61 | 6/27 | 0/17 | 3/13 | 115 |
| 15-Nov | 63/258 | 6/29 | 21/43 | 15/68 | 14/58 | 5/34 | 0/8 | 2/12 | 0/6 | 63 |
| 15-Dec | 67/275 | 6/28 | 15/40 | 18/73 | 12/65 | 10/36 | 5/21 | 0/7 | 1/5 | 67 |
| 16-Jan | 61/234 | 6/23 | 12/27 | 15/67 | 17/50 | 8/41 | 3/14 | 0/7 | 0/5 | 61 |
| 16-Feb | 70/220 | 5/12 | 14/31 | 28/76 | 11/47 | 8/22 | 1/13 | 3/14 | 0/5 | 70 |
| 16-Mar | 64/191 | 5/8 | 8/23 | 17/64 | 18/46 | 12/29 | 2/10 | 1/9 | 1/2 | 64 |
| 16-Apr | 38/120 | 0/3 | 5/10 | 13/42 | 10/31 | 2/16 | 7/14 | 0/3 | 1/1 | 38 |
| Total | 848/3161 | 61/215 | 170/418 | 261/941 | 182/724 | 109/455 | 41/217 | 14/121 | 10/70 | 848/3161 |
|  |  | 28.4% | 40.7% | 27.7% | 25.1% | 24.0% | 18.9% | 11.6% | 14.3% | 26.8% |
